# Hydrocele of the Canal of Nuck: A Review

**DOI:** 10.7759/cureus.23757

**Published:** 2022-04-02

**Authors:** Nattawut Keeratibharat, Jirapa Chansangrat

**Affiliations:** 1 Surgery, Institute of Medicine, Suranaree University of Technology, Nakhon Ratchasima, THA; 2 Radiology, Institute of Medicine, Suranaree University of Technology, Nakhon Ratchasima, THA

**Keywords:** hernia, inguinal mass, the canal of nuck, hydrocele of the canal of nuck, hydrocele

## Abstract

Canal of Nuck abnormality is a rare surgical condition. The pathologies are mostly encountered in young girls, less than five years of age. The incidence is even less in adults. Various pathologic conditions related to the failure of processus vaginalis obliteration can occur, involving herniation of intraabdominal structures including intestinal and genital contents such as the uterus, fallopian tube, and ovary and hydrocele of the canal of Nuck.

According to its rarity, hydrocele of canal of Nuck is often misdiagnosed for common groin masses. This review summarizes and simplifies embryology, the pathophysiology of the canal of Nuck abnormalities, imaging findings, and treatment options with emphasis on the hydrocele.

## Introduction and background

Anton Nuck described the canal of Nuck for the first time in 1691 [1]. Normally, the processus vaginalis closes during the first year of life. Failure of the closure results in various pathologies presented as groin lumps. These clinical presentations need to be distinguished from other groin masses. Understanding the embryology and pathophysiology of these conditions could help in the differential diagnosis. Awareness of this entity is critical in order to get an appropriate diagnosis from imaging exams and prevent unneeded biopsy or surgical intervention. The pathogenesis of congenital abnormality, diagnostic modality and treatment are summarized in this review.

## Review

Embryology

The gubernaculum and the processus vaginalis, two important components in the development of the inguinal canal, are both involved. The gubernaculum is a fibromuscular structure that develops between eight and 12 weeks in a fetus [2]. The structure is connected to the inferior pole of the growing gonad and extends down to the groin area. In males, the distal gubernaculum continues to grow to enable the testes to descend into the scrotum. Because of the lack of influence from androgen, the gubernaculum in females stops growing, allowing the gonadal content in the pelvis to be retained in the pelvis. It is attached to the uterine cornua and develops into the ovarian suspensory ligament above the site of connection between two structures. The distal part forms the round ligament that descends into the inguinal canal and extends to the labia majora. 

**Figure 1:**
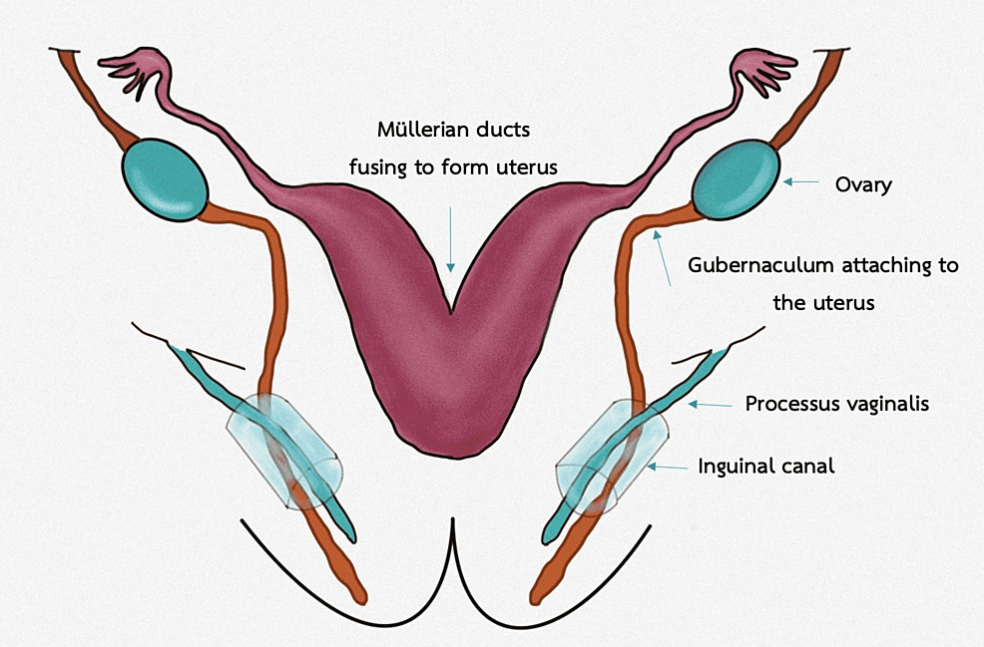
Embryology during the third month of gestation Illustration created by the authors.

**Figure 2:**
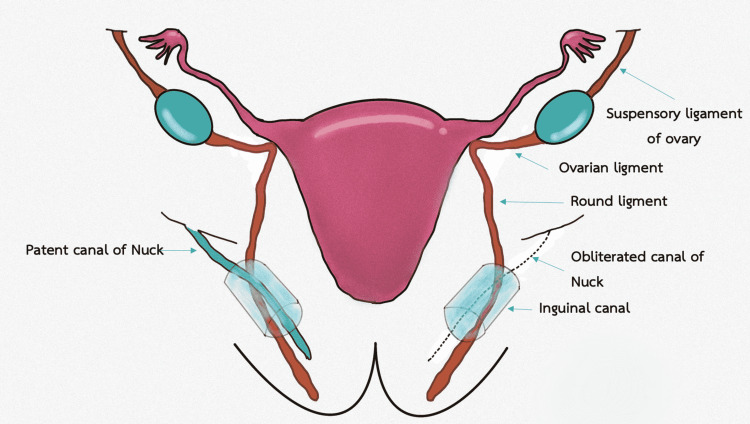
Embryology during the fourth month of gestation Illustration created by the authors.

The processus vaginalis is an invagination of the parietal peritoneum that enters the deep inguinal ring before the gubernaculum. Within the inguinal canal, it lies medial to the gubernaculum [3]. The processus vaginalis develops in the first trimester. In males, it facilitates the gubernaculum to create a dilated passage for the testicular descending to the scrotum. The canal of Nuck refers to the section of the processus vaginalis that is located in the inguinal canal in females [4]. Figure 1 and Figure 2 show illustrations of embryology during the third and fourth months of gestation, respectively.

The pathogenesis of hydrocele of the canal of Nuck and related abnormalities

Normally, in humans, from the eighth month of pregnancy to the first year of life, the canal of Nuck completely disappears in a craniocaudal axis. Failure of the obliteration of the canal of Nuck leads to various pathologies. Complete failure of closure results in a patent canal of Nuck. Abnormalities following this condition include communicating hydrocele and hernia. If the gubernaculum fails to adhere to the uterine cornua, the ovary might descend via the inguinal canal [5]. Herniation of the uterus into the ovary has been seen in certain cases [6-8]. A uterine herniation is almost typically associated with the presence of a fallopian tube or ovarian hernia [9]. Herniation of an ovary could lead to incarceration, strangulation, or ovarian torsion which requires emergency treatment.

Incomplete closure of the canal of Nuck leads to the formation of hydrocele. When the proximal portion of the processus vaginalis closes, and the distal portion is still patent, the processus vaginalis which is lined by mesothelial cells will secrete fluid that accumulates in the potential space. The hydrocele can be elongated, or sausage-shaped which follows a narrow configuration of the inguinal canal. As the disproportion between production and absorption of the secreted fluid continues, the hydrocele becomes more rounded [2]. Classification of hydrocele of the canal of Nuck has been described [10] as follows:

Type 1: An encysted hydrocele is a kind of cyst that develops as a consequence of partial obliteration of just the proximal portion of the canal of Nuck. Typical clinical presentation is a nonreducible, painless mass whose volume remains unchanged when the Valsalva maneuver is performed [11]. When there is an imbalance between fluid secretion and absorption, which can occur as a result of trauma, inflammation, or lymphatic drainage impairment, the cyst can abruptly grow in size [12]. Within a closed cyst, an infection might develop following a slight trauma or intracystic hemorrhage. The patient may complain of a painful mass that is mistaken for an incarcerated hernia [13].

Type 2: A communicating hydrocele. This pathology occurs from the patency of the canal of Nuck. It appears as a non-tender reducible mass that may only arise after performing the Valsalva maneuver or standing.

Type 3: A combined type. The lesion has an encysted inferior section in the inguinal canal and labia majora, as well as an upper intraabdominal portion. The hydrocele is compressed by the deep inguinal ring, creating an hourglass appearance.

Clinical manifestation

A hydrocele of the canal of Nuck may manifest clinically as either a painless or a painful fluctuant inguinal mass, with no accompanying nausea or vomiting. As a result, it is difficult to identify this entity only based on clinical signs. When executing the Valsalva maneuver, this mass normally extends to the labia majora and does not enlarge [14]. In case the mass is not reducible and, if it is big enough, it may be transilluminated. As long as the peritoneal evagination is patent, it creates an opening for the development of an indirect inguinal hernia. The anatomy of a hydrocele of the canal of Nuck is formed by partial proximal obliteration of the processus vaginalis, which leaves the distal section of the processus vaginalis intact.

In women, inguinal swelling should be differentiated from an indirect inguinal hernia, tumors (lipoma, leiomyoma, and sarcoma), cyst, abscess, and lymphadenitis [12]. The canal of Nuck should be differentiated from an inguinal hernia in most cases, and a hernia should be highly suspected when bowel noises are heard from the mass. Unless the hernia component co-exists with the bowel as the content, there may be no signs of intestinal obstruction. Hernias are often noticed when the patency is big enough to enable the bowel or omentum to protrude into the canal [1].

Imaging

Ultrasound

**Figure 3:**
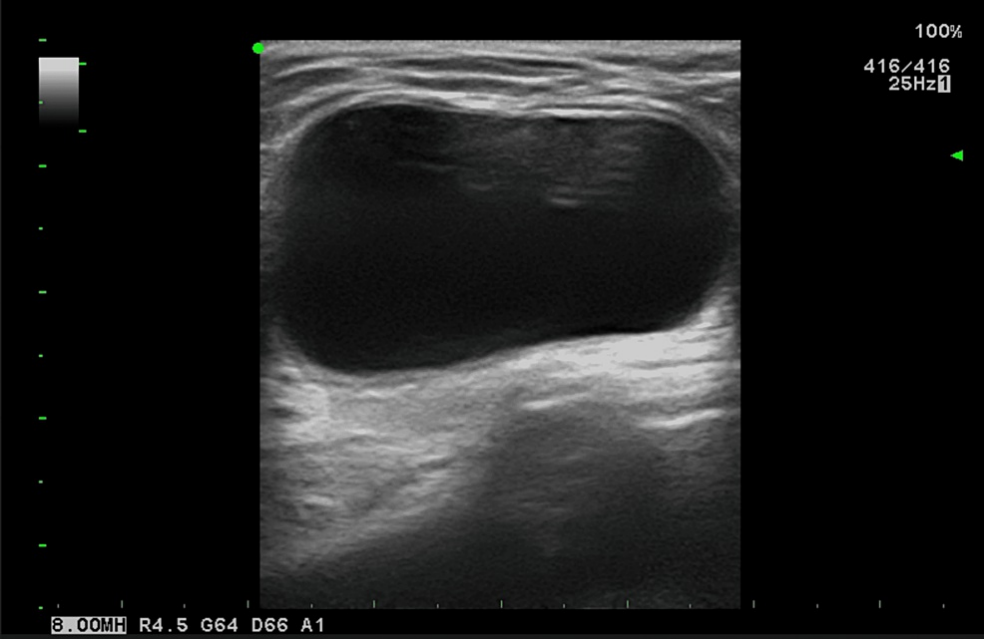
Ultrasound of authors' patient shows echogenicity with a posterior acoustic enhancement of hydrocele of the canal of Nuck

For initial imaging of canal of Nuck abnormalities, ultrasound is the preferred method [15]. The modality shows superiority in accessing superficial structures with high-resolution images. A hydrocele of the canal of Nuck usually appears as a well-defined anechoic lesion with posterior acoustic enhancement [9,16]. Figure 3 shows a typical ultrasound finding of a hydrocele of the canal of Nuck. The lesion might appear hypoechoic or demonstrate low-level echoes due to high protein content. When complication of the hydrocele such as infection or hemorrhage occurs, the lesion probably shows a complex appearance which are echogenic content such as thickened wall, or internal septation. Due to the existence of a tiny, thin-walled cyst inside that is linked to the larger cyst that moves in reaction to the pressure applied by the transducer, the look of a "cyst-in-cyst" may be seen. A cyst-in-cyst feature may be mistaken for a follicular ovary, which likewise has thin vascularized parenchyma surrounding it. These findings need to be distinguished from incarcerated bowel loop by demonstration of continuity of content with the intraperitoneal cavity in the latter condition. There should be no color flow in the lesion when using Doppler ultrasound.

Magnetic Resonance Imaging (MRI)

When ultrasonography is inconclusive, an MRI is used to obtain additional information about the herniated structures [2,17]. There is no radiation associated with this imaging modality. Its larger field of view enables a more precise anatomical relationship between the pathology in the canal of Nuck and intraperitoneal structures. The canal of Nuck hydrocele is often hypointense on T1-weighted imaging and hyperintense on T2-weighted images. Faint internal septation can be found if there is a coexistence of infectious or inflammatory process [18]. An MRI can also help distinguish pathologies of the canal of Nuck from other soft tissue tumors that can present as a palpable inguinal mass. However, patients need to stay still for the long scanning time which is difficult in the case of pediatric patients.

Computed Tomography (CT)

A CT scan is not the first imaging modality to choose when accessing groin mass due to its radiation. However, due to its short scanning time, high availability, and the possibility of an uncertain diagnosis of inguinal hernia and other groin mass, a CT scan is commonly performed in an adult population.

**Figure 4:**
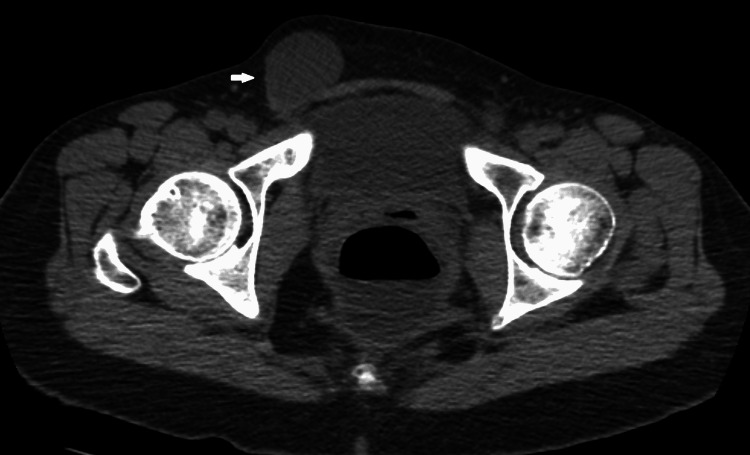
Plain axial CT scan of authors' patient shows comma-shaped hydrocele that contains fluid attenuation (arrow)

**Figure 5:**
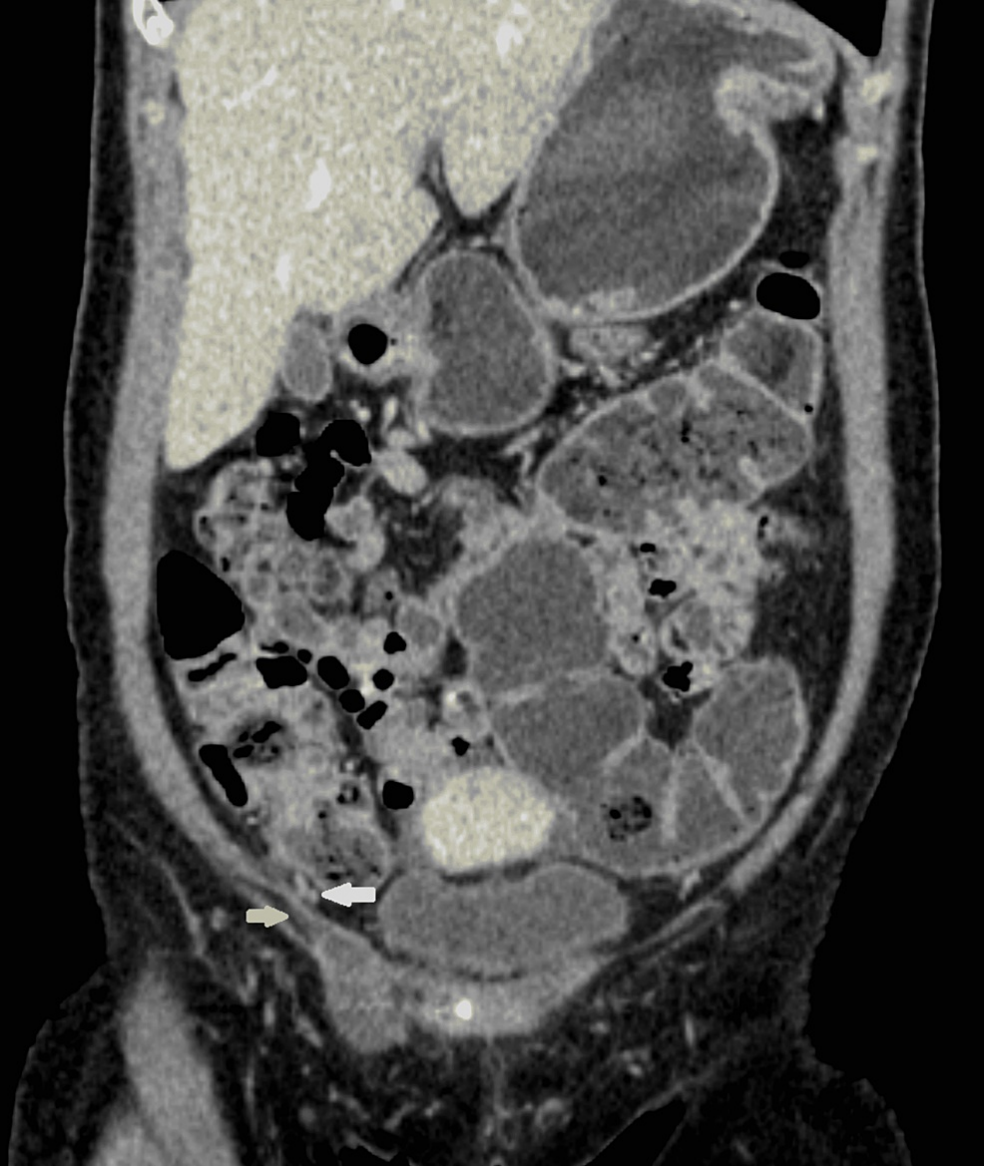
Coronal CT scan of authors' patient in venous phase shows the proximal origin of hydrocele of the canal of Nuck (yellow arrow) lateral to inferior epigastric vessels (white arrow)

Another circumstance is when a patient presented with nonspecific abdominal pain at the emergency department, a CT scan would be requested to evaluate the cause of the abdominal pain, and pathology of the canal of Nuck would be incidental findings. When the inguinal canal is simple, a well-defined low-density collection may be seen on CT imaging, which can be used to quantify fluid attenuation. Figure 4, and Figure 5 show the typical CT of a hydrocele of the canal of Nuck. When the collection is complicated, such as when there are septations or proteinaceous/hemorrhagic contents, the collection is somewhat above fluid attenuation. On CT, neither simple nor complex cysts exhibit any contrast enhancement [2].

Treatment

**Figure 6:**
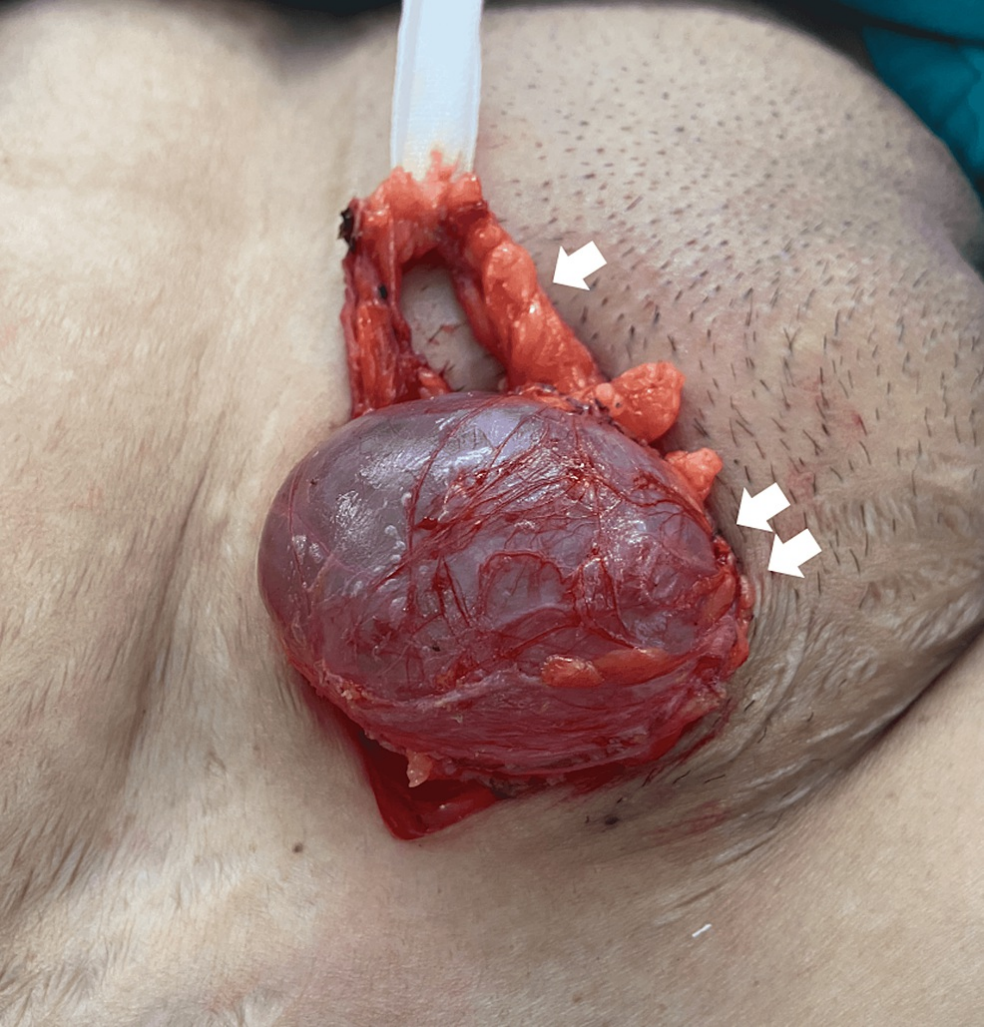
Intraoperative picture by the authors - a hydrocele of the canal of Nuck (two arrows). The round ligament was encircled (one arrow).

**Figure 7:**
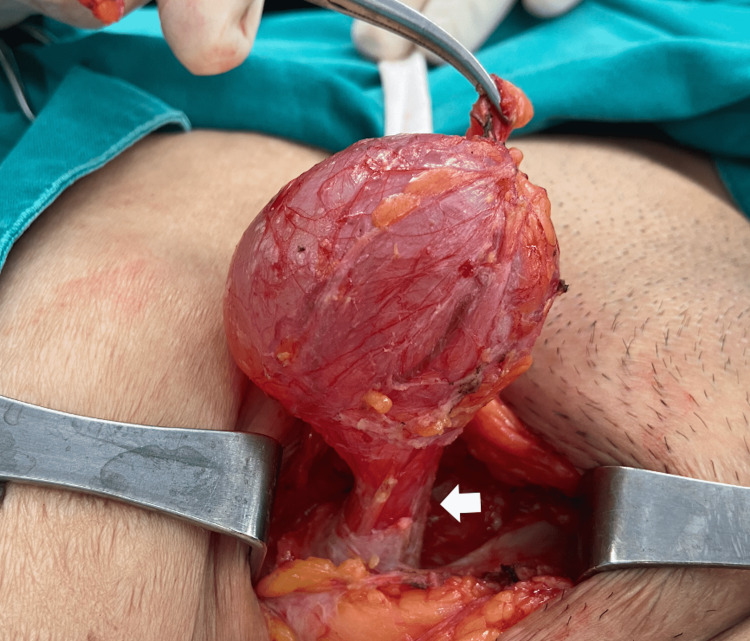
Intraoperative picture of a hydrocele of the canal of Nuck taken by the authors. This picture shows the obliteration of proximal processus vaginalis (arrow).

Acute or chronic symptoms, as well as hydrocele infections, are possible [13,19,20]. Even though a great number of hydroceles are reported, most do not warrant urgent surgical intervention. However, a few do due to infections, tumors, or herniation into the canal of Nuck. The surgical treatment for a cyst of the Canal of Nuck is excision of the cystic structure with concomitant closure of the inguinal defect, with or without mesh [21-23]. Recurrences may be reduced by surgically closing the prosessus vaginalis and eliminating the cyst. The extent of the condition, precision of the preoperative diagnosis, the presence of an inguinal hernia, and other factors all determine the most appropriate surgical procedure. If an inguinal hernia is diagnosed at the same time, a hernia repair with or without mesh insertion may be done simultaneously. An extra vulva repair may be advised in situations with mass extension to the labia majora. [24]. Intraoperative pictures of a hydrocele of the canal of Nuck are shown in Figure 6 and Figure 7.

Although there have been instances of laparoscopic excision of a canal of Nuck cyst in recent years, individuals with a canal of Nuck cyst with an indirect inguinal hernia might contemplate laparoscopic surgery [25,26]. Concurrent correction of the inguinal hernia is feasible. If it's only a simple cyst in the canal of Nuck, the laparoscopic excision will almost certainly result in an expansion of the inner ring, which will need the placement of a patch to fasten. [27]. Furthermore, due to the restricted view of the inguinal anatomy during laparoscopic cystic excision, distal end cyst removal is a challenging procedure that requires expert skill and experience [28]. If laparoscopy confirms that the patient has a pure cyst of the canal of Nuck at this time, the anterior resection may be done without hesitation, perhaps shortening the operative time [29]. Thus, laparoscopy may be the most effective tool for identifying the probable point of weakness and internal organ confinement in the peritoneal cavity due to increasing intra-abdominal pressure of the pneumoperitoneum [19,30,31].

As a consequence, surgeons must choose the appropriate approach for the diagnosis and treatment of a canal of Nuck cyst depending on the woman's age, concomitant with an inguinal hernia, and whether the patient desires to get pregnant in the future. The laparoscopic technique in combination with the anterior approach is the most exact diagnostic procedure and the shortest operation for the treatment of a pure cyst of the canal of Nuck. It may be the most effective treatment option for young women of reproductive age who do not have a hernia [32]. Furthermore, cyst aspiration may help ease symptoms but is not suggested as a definite treatment [33].

Discussion

The canal of Nuck hydrocele in the setting of groin mass is a rare occurrence that must be correctly identified in terms of terminology once an accurate diagnosis is achieved. According to 19th century literature, such cases were commonly misconstrued and hence identified and treated as common inguinal or femoral hernias [34]. For decades, there were just a few cases when this entity was mentioned in individual case reports. Initially, the limited case studies focused only on diagnosis and not on surgical treatment [10,35]. Only one case report has addressed the adult entity of Nuck's hydrocele. There are still no documented case series of adult females with this inguinal condition.

There is a possibility of several different masses in the female inguinal area. In summary, hernia, lymphadenopathy, abscess, Bartholin's cyst, and hematoma are all possible diagnoses for this condition. [36]. There have also been instances of endometriosis in the canal of Nuck [22,37]. Similar results are possible in the canal of Nuck, with the most frequent being isolated hydroceles or hernias [2]. Furthermore, disease in the canal of Nuck may be more prevalent than previously thought and should be included in the differential diagnosis of groin discomfort [38].

For the reason that it is inexpensive and widely available, ultrasonography may be used to get initial imaging information, whereas magnetic resonance imaging (MRI) can be used for more complicated issues and additional investigation [38,39]. Sonographic findings typically show an extended mass containing anechoic fluid in the form of a well-defined anechoic lesion [40]. Omental fat or intraperitoneal organs may be present in a hernia [38]. A Valsalva maneuver may occasionally modify the content of a hernia while leaving an encysted hydrocele untouched [41]. As previously indicated, MRI may be used to evaluate ambiguous sonographic data, especially when a herniation is suspected [38].

A hydrocelectomy would be performed first, followed by a hernioplasty. Transabdominal preperitoneal repair (TAPP) and Lichtenstein hernioplasty are comparable and they may be used equally as treatments. [19]. A comparison of the two techniques is anticipated to provide findings similar to those reported in a meta-analysis of inguinal hernia in men, and as a result TAPP was shown to be related to reduced persistent inguinal pain than a Lichtenstein repair. [42]. Furthermore, the laparoscopic total extraperitoneal hernia repair (TEP) approach is a viable option [19]. Both TAPP and TEP are anticipated to provide equivalent results, with TAPP having benefits in terms of shorter operation time and lower conversion rates [43]. Moreover, it is well known that finding anatomic landmarks when utilizing the TEP technique is more difficult than when using the TAPP approach, and hence this approach is not appropriate for exploration [44]. Ultrasound-guided cyst aspiration might be used to ease patient suffering temporarily, especially in unfit patients who are unable to undergo surgery [12].

## Conclusions

The hydrocele of the canal of Nuck is rarely discussed in depth in surgical and gynaecological textbooks, and it remains an unfamiliar condition for clinicians. In certain circumstances, this issue is sometimes misidentified as an inguinal hernia or an abscess. Ultrasonography is a useful and initial modality for distinguishing the hydrocele of the canal of Nuck from other conditions. In the event of a hernia, the treatment of choice is hydrocelectomy, followed by hernioplasty, both of which are surgical procedures.
